# Health Care Needs and Support for Patients Undergoing Treatment for Prosthetic Joint Infection following Hip or Knee Arthroplasty: A Systematic Review

**DOI:** 10.1371/journal.pone.0169068

**Published:** 2017-01-03

**Authors:** Setor K. Kunutsor, Andrew D. Beswick, Tim J. Peters, Rachael Gooberman-Hill, Michael R. Whitehouse, Ashley W. Blom, Andrew J. Moore

**Affiliations:** Musculoskeletal Research Unit, School of Clinical Sciences, University of Bristol, Bristol, United Kingdom; Cardiff University, UNITED KINGDOM

## Abstract

**Background:**

Hip and knee arthroplasty are common interventions for the treatment of joint conditions, most notably osteoarthritis. Although many patients benefit from surgery, approximately 1% of patients develop infection afterwards known as deep prosthetic joint infection (PJI), which often requires further major surgery.

**Objective:**

To assess support needs of patients undergoing treatment for PJI following hip or knee arthroplasty and to identify and evaluate what interventions are routinely offered to support such patients.

**Design:**

Systematic review

**Data sources:**

MEDLINE, EMBASE, Web of Science, PsycINFO, Cinahl, Social Science Citation Index, The Cochrane Library, and reference lists of relevant studies from January 01, 1980 to October 05, 2016.

**Selection criteria:**

Observational (prospective or retrospective cohort, nested case-control or case-control) studies, qualitative studies, or clinical trials conducted in patients treated for PJI and/or other major adverse occurrences following hip or knee arthroplasty.

**Review methods:**

Data were extracted by two independent investigators and consensus was reached with involvement of a third. Given the heterogeneous nature of study designs, methods, and limited number of studies, a narrative synthesis is presented.

**Results:**

Of 4,213 potentially relevant citations, we identified one case-control, one prospective cohort and two qualitative studies for inclusion in the synthesis. Patients report that PJI and treatment had a profoundly negative impact affecting physical, emotional, social and economic aspects of their lives. No study evaluated support interventions.

**Conclusion:**

The findings demonstrate that patients undergoing treatment for PJI have extensive physical, psychological, social and economic support needs. The interpretation of study results is limited by variation in study design, outcome measures and the small number of relevant eligible studies. However, our review highlights a lack of evidence about support strategies for patients undergoing treatment for PJI and other adverse occurrences following hip or knee arthroplasty. There is a need to design, implement and evaluate interventions to support these patients.

**Systematic Review Registration:**

PROSPERO 2015: CRD42015027175

## Introduction

Joint arthroplasty is a common surgical procedure, with over 185,000 primary hip and knee arthroplasties performed in England, Wales and Northern Ireland in 2014 [1, 2]. After total hip or knee arthroplasty, about 1% of patients develop an infection of their new, artificial joint [3, 4]. This is known as prosthetic joint infection (PJI). When PJI is identified, further major operations are commonly needed to clear the infection, which reduces the need for subsequent joint removal or even limb amputation. Surgical treatment involves extensive debridement with either prosthesis retention or surgical revision with a new prosthetic joint inserted either immediately (one-stage procedure) or after a delay, typically between 3 and 6 months (two-stage procedure). If delayed, a temporary implant or “spacer”, often impregnated with antibiotics may be fitted; however further complications including spacer dislocations and femoral fractures are common with the use of such spacers [5]. Surgical treatments are used in conjunction with the use of powerful intravenous and oral antibiotics. After revision surgery has been performed, reinfection occurs within two years in around 8% of patients requiring further major surgery, lifelong antibiotic suppression or amputation [6–8]. Although PJI affects a small percentage of patients, hip and knee replacements are common and PJI is considered to be one of the most devastating complications associated with this procedure [9]. It typically presents with a leaking wound or onset of fever, pain, swelling, worsening joint pain and restriction of movement and may develop into systemic sepsis [10, 11]. If left untreated, the condition can cause death [12].Research evidence on the treatment of prosthetic joint infection has focussed on the clinical effectiveness of different types of revision surgery [6–8] while there appears to be less focus on the impact on patients and their support needs. During the hospital discharge process, patients receiving orthopaedic interventions, including total hip and knee replacements, report a negative mental outlook, functional and activity limitations, pain and loss of independence [13]. After a range of hospital admissions, individualised discharge planning strategies may lower the risk of readmission and improve patient satisfaction [14]. Post-discharge support for patients treated with revision surgery is not currently included in clinical guidelines [15, 16]. However, recognising their importance in other conditions with a high risk of a poor outcome such as congestive heart failure [17] and chronic obstructive pulmonary disease, [18] there is a pressing need to identify the support needs of patients with PJI and to provide effective support. Such information can be used to design or improve services that may deliver benefit for patients.

The Medical Research Council (MRC) guidance on developing complex interventions suggests that the first step in the development process is “to identify what is already known about similar interventions and the methods that have been used to evaluate them. If there is no recent, high quality systematic review of the relevant evidence, one should be conducted and updated as the evaluation proceeds” [19]. In line with the MRC guidance we used a systematic review approach; the primary aim of which was to assess support needs and care pathways for patients undergoing treatment for PJI. However, given that the treatment burden and support needs of these patients may share similarities with other adverse occurrences following joint replacement, for example recurrent dislocation, we aimed to include other major adverse occurrences following hip and knee arthroplasty that would also have a high chance of revision surgery and a similar impact on the patient. Our search strategy was therefore widened to include joint dislocation, fracture, pseudotumour, prosthesis failure, aseptic loosening and septic arthritis. This would allow us to define the extent of unmet needs for patients with PJI after hip and knee arthroplasty and to evaluate existing support interventions for patients with PJI or conditions with a similar impact that might prove useful in the development of a new care pathway. Finally, we also sought to identify gaps in the available literature.

## Methods

### Data sources and search strategy

This review was conducted using a predefined protocol, which was registered in the PROSPERO prospective register of systematic reviews (CRD42015027175) and in accordance with PRISMA guidelines [20] (**S1 Appendix**). We systematically searched MEDLINE, EMBASE, Web of Science, PsycINFO, Cinahl, Social Science Citation Index, and the Cochrane Library from 1980 to October 05, 2016 for observational (prospective cohort, nested case-control, case-control, and retrospective cohort) studies, qualitative studies, and clinical trials that have reported on the support needs and interventions for patients being treated for PJI or other major adverse occurrences following joint arthroplasty. The computer-based searches combined free and MeSH search terms and combination of key words related to support and care pathways (including physical rehabilitation, counselling, peer support, improved information) and adverse outcomes (including periprosthetic joint infection, dislocation, aseptic loosening) without language restriction. We adapted the MEDLINE search strategy to the other databases using the appropriate controlled vocabulary. Reference lists of retrieved articles were manually scanned for relevant additional studies missed by the electronic search. Details on the search strategy are provided in **S2 Appendix**.

### Eligibility criteria

Studies were eligible if they reported on i) support needs (patient identified needs associated with the outcomes of joint arthroplasty) and interventions (including physical rehabilitation, counselling, peer support, improved information) for ii) adult patients (≥ 18 years) iii) undergoing treatment for PJI or other major adverse occurrences (joint dislocation, fracture, pseudotumour, prosthesis failure, aseptic loosening, and septic arthritis) iv) following joint arthroplasty or revision surgery.

### Data extraction and quality assessment

One reviewer (S.K.K.) initially performed screening of abstracts and potentially relevant articles were acquired. We then retrieved full text articles of these studies to determine whether they met all inclusion criteria, which was conducted independently by two reviewers (S.K.K. and A.J.M.). Disagreements and uncertainties were resolved by discussion, with the involvement of a third reviewer (A.D.B) if necessary. A standardized predesigned data collection form was used for data extraction. Data were abstracted, where available, on study design, baseline population characteristics, geographical location, year of recruitment, percentage of males, mean age, description of complication after joint arthroplasty, treatment for complication, and number of participants. For studies with a prospective cohort design, methodological quality was assessed based on the Methodological Index for Non-Randomised Studies (MINORS), a validated instrument that is designed for assessment of methodological quality of non-randomised studies in surgery[21]. For non-comparative studies, it uses eight pre-defined domains namely: a clearly stated aim, inclusion of consecutive patients, prospective collection of data, endpoints appropriate to the aim of the study, unbiased assessment of the study endpoint, follow-up period appropriate to the aim of the study, loss to follow-up less than 5%, and prospective calculation of the study size. For each item, MINORS assigns 0 for not reported, 1 for reported but inadequate, or 2 for reported and adequate. The global ideal score is 16. For the study with a case-control design, study quality was assessed based on the nine-point Newcastle–Ottawa Scale (NOS) [22] using three pre-defined domains namely: selection of participants (population representativeness), comparability (adjustment for confounders), and ascertainment of outcomes of interest. The NOS assigns a maximum of four points for selection, two points for comparability, and three points for outcome. Nine points on the NOS reflects the highest study quality. A critical appraisal of included qualitative studies was conducted using the Specialist Unit for Review Evidence (SURE) Checklist [23]. This tool comprises of 10 questions that address broad issues such as clearly defined research questions, methodology, and validity of results. We considered the characteristics of each study in relation to each of these questions. Based on these characteristics the articles were classified as ‘fully address SURE questions’ if they attended to *all* of the questions, ‘mainly address SURE questions’ if they attended to *most* of the questions, or ‘partially address SURE questions’ if they attended to *some* of the questions.

### Data synthesis and analysis

It was not possible to calculate summary measures across studies given the limited number of studies, diversity of the study designs, and the heterogeneous nature of the outcomes. The characteristics of each study were summarised in a table. A narrative synthesis with separate results from each study was therefore performed. For the above reasons, subgroup and sensitivity analyses, and exploration of heterogeneity could not be conducted.

## Results

### Study identification and selection

**Fig 1:**
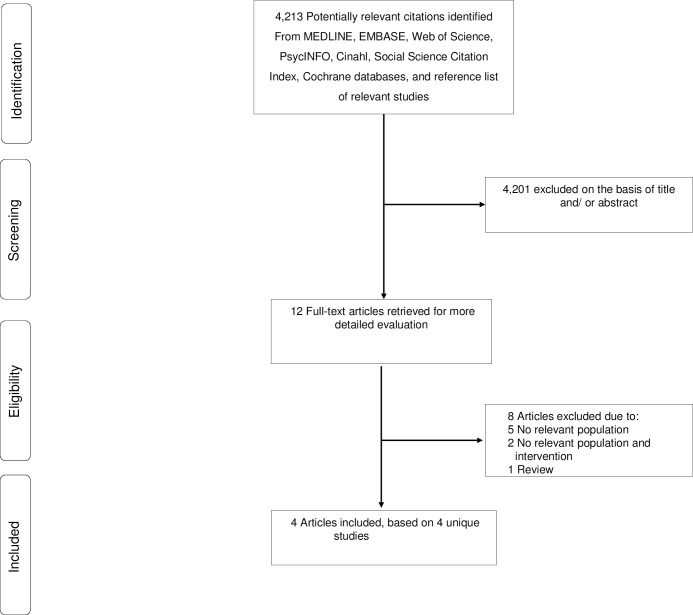
PRISMA Flow Diagram.

Our initial search of databases and manual screening of reference lists of relevant studies identified 4,213 potentially relevant citations. After screening based on titles and abstracts, 12 articles remained for further evaluation. Following detailed assessments by two reviewers, eight articles were excluded (**S3 Appendix**), while four studies met the inclusion criteria and were included in the review (**Fig 1**).

### Study characteristics and quality

**Table 1** summarises the key characteristics of the four studies included in the review [24–27]. The studies were conducted in the USA, Australia, Sweden and the United Kingdom. Two studies employed qualitative designs, one was a prospective cohort and the other was a case-control study. All included studies were judged to be of adequate (moderate to high) methodological quality.

**Table 1:** Summary characteristics of included studies.

Lead author, publication date	Location	Baseline year of study	Study design	Population	Males (%)	Mean/median age (years)	Adverse outcome after joint arthroplasty	Treatment for adverse outcome	Number of participants/joints	Study quality
Barrack, 2000	USA	NR	Prospective cohort	Patients who underwent septic and aseptic revision after total knee arthroplasty	NR	69.2 (68.5 for septic revision and 69.5 for aseptic revision)	Infection, aseptic component loosening, progressive osteolysis, and component instability	Two-stage revision	125 patients comprising of 127 knees (28 infected and 99 not infected)	11
Cahill, 2008	Australia	2003–2004	Case-control	Patients with and without a complication of deep infection after TJA	46.9 (43.5 for uncomplicated and 52.9 for complicated)	71.0 (71.0 for uncomplicated and 71.0 for complicated)	Deep infection	Washout/antibiotic; two-stage revision; and excision	96 patients (62 uncomplicated and 34 complicated)	5
Andersson, 2010	Sweden	NR	Qualitative descriptive	Participants diagnosed with deep SSI	64.3	NR	Deep SSI	NR*	14	Fully address SURE questions
Moore, 2015	United Kingdom	2014	Qualitative semistructured interviews	Patients who had undergone surgical revision treatment for PJI	63.2	73.2	PJI	One- and two-stage revision	19	Fully address SURE questions

*, indicated that patients had received and completed active treatment; NA, not applicable; NR, not reported; PJI, prosthetic joint infection; SSI, surgical site infection; TJA, total joint arthroplasty.

### Support needs

Barrack and colleagues prospectively followed 125 patients who had a revision total knee arthroplasty for a minimum of two years and compared patient satisfaction, social functioning, and functional outcomes between a group revised for infection and a group revised for reasons other than infection [25]. Patients in the two groups expressed an equal degree of satisfaction with their treatment; however, patients revised for infection reported lower functional outcomes and were less likely to return to their normal activities of daily living. Cahill and colleagues compared patient satisfaction, function, and health-related quality of life among patients with uncomplicated joint replacements and joint replacements complicated by deep infection [24]. Questionnaires were administered to patients by phone and during face-to-face interviews. Treatment options for patients with infection included washout with antibiotics, two-stage revision, and excision. Overall, patients in the group with deep infection had poorer functional and health-related quality-of-life outcomes compared to the group without deep infection. Infection greatly impacted on their physical and social functioning, their ability to live independently and to perform activities of daily living. In their qualitative study, Andersson and colleagues aimed to describe patients’ experiences of acquiring a deep surgical site infection (SSI) [26]. They analysed data from 14 open-ended interviews with participants who were diagnosed with a deep SSI and had completed treatment. Of the 14 participants interviewed 12 acquired infection after lower limb operations, 1 after coronary bypass and 1 after abdominal hysterectomy. The overall results demonstrated that patients with infection and undergoing treatment experienced negative and often persistent changes in their lives, notably in relation to pain, social isolation, sense of security, physical, economic and social wellbeing. The study also showed inconsistencies in care and absence of adequate patient-healthcare professional relationships. The authors called for strategies to support patients undergoing treatment for infection and the need to involve patients in their care. In their qualitative interview study, Moore and colleagues aimed to characterise the impact and experience of PJI and treatment on patients, including a comparison of one- and two-stage revision treatment [27]. They analysed data from 19 interviews with participants with PJI (one-stage revision n = 9, and two-stage revision n = 10). Infection and treatment occurred over periods ranging from 12 months to more than 10 years. The number of revision operations received ranged from one to 15. Patients described loss of physical function and mobility in relation to the infection and the treatment. Those who received a two-stage revision reported additional challenges related to the interim period during which they had no prosthetic hip joint when either a spacer was fitted or not. Those left without a spacer faced considerable physical and psychological difficulties. One patient reported considering suicide during this period. Other patients who were fitted with a spacer for a period ranging from 10 weeks to 14 months, reported complications associated with the spacer including pain, immobility, fracture and dislocation which caused further physical and psychological trauma. The overall impact of PJI and treatment had a profound impact on physical, emotional, social and economic aspects of patients’ lives. Patients also reported financial and employment difficulties resulting from PJI. Support needs identified by participants included individualised physiotherapy, psychological support and counselling and allied professional aftercare (osteopathy, podiatry), home care and support in caring for others. Patients also identified a need for additional information about the interim period of a two-stage revision.

### Support interventions

No study evaluated any support interventions (including physical rehabilitation, counselling, peer support, improved information) for patients being treated for PJI or other major adverse occurrences following joint arthroplasty.

### Excluded studies

The list of excluded studies after full text evaluation is provided in **S3 Appendix**. The majority of these articles focused on the impact of preoperative clinical pathways on major outcomes after joint arthroplasty. One was a quantitative review of 22 studies that evaluated the impact of clinical pathways compared to standard medical care, on postoperative complications, quality of care, and direct costs after hip and knee joint arthroplasties.

## Discussion

### Key findings

Using a systematic review approach, we have assessed evidence on the support needs and support interventions for patients being treated for PJI and other major adverse occurrences following joint arthroplasty. In a comprehensive search of several databases, only four studies were eligible and were included in the review. While all included studies focussed on support needs for patients with PJI, one study also included patients with adverse outcomes such as aseptic component loosening and component instability [25]. Overall, our findings based on the limited number of studies, indicate that infection has a considerable impact on all aspects of patients’ lives, and when undergoing treatment they can experience feelings of isolation and insecurity, and an inability to live independently. Cahill and colleagues noted that more psychological and social support was needed for this group of patients, given the negative impact on their social functioning, and that the impact on mental health is notable. This group of patients rated their overall quality of life as poor with 12% of patients rating their current situation as equivalent to or worse than death [24]. Andersson and colleagues reported inconsistencies in care and the lack of an adequate patient-healthcare professional relationship in 14 patients who had acquired deep surgical site infection and had completed treatment [26]. The study authors stressed the need for healthcare professionals to develop strategies to support patients undergoing treatment for infection and also to involve patients in their care. Moore and colleagues found that PJI and its treatment had a profoundly negative impact on many aspects of patients’ lives and patients identified a clear need for additional physical, psychological, social care and economic support both during and after treatment. The authors found that two-stage revision treatment was associated with considerable physical and psychological burden and further complications. No studies were identified that evaluated existing support interventions (including physical rehabilitation, counselling, peer support, improved information) for patients being treated for PJI or other major adverse events after joint arthroplasty.

### Implications of our findings

Our results are relevant and provide new insight into the limited evidence available regarding support needs and provision for patients undergoing treatment for PJI and other adverse events after joint arthroplasty. Our review clearly identifies gaps in the literature. Although for many people, hip and knee arthroplasty are highly successful and cost-effective interventions for alleviating pain and disability associated with advanced joint disease, arthroplasty is also associated with uncommon but serious complications, such as PJI [28, 29]. Patients who undergo treatment with one-stage or two-stage revision surgery may experience sustained psychological distress both during and after treatment, and further pain, disability, and even poorer subsequent quality of life [24, 27]. Prosthetic joint infection and other complications of joint arthroplasty constitute a major burden for patients and health systems as a whole and with increasing numbers of operations being performed, the number of infections will likely remain significant [30]. This review shows clearly there is a need for the development, evaluation and implementation of effective care pathways that address the physical and psychological trauma associated with complications after joint arthroplasty and their treatments and to support patients at each stage of their diagnosis, treatment and recovery.

### Strengths and limitations

This is the first review to identify a lack of evidence on support needs and interventions for patients undergoing treatment for prosthetic joint infection or other major adverse occurrences following hip or knee arthroplasty. With a comprehensive literature search across multiple databases, we identified 4 studies which provide evidence on the support needs of patients being treated for PJI or other major adverse events following hip or knee replacement and appropriate support interventions. The review was limited by the small number of heterogeneous studies that were identified. While the evidence is limited the general objective of a systematic review is to identify best evidence and to evaluate it where it exists, as well as to identify important gaps within the research evidence. These studies highlight the considerable negative impact that these adverse events have on patients and identify their unmet needs, while the lack of evidence also suggests there is a lack of service provision for this patient population that needs to be addressed.

The eligible studies from the review were conducted in a diverse group of countries, which may affect the generalisability of the findings; however, surgical practices around hip and knee arthroplasty in these countries is similar, and our findings suggest that PJI has a similar negative impact on patients in each of these countries. In addition, none of the studies in each of these countries evaluated existing support interventions for patients being treated for PJI or other major adverse events after joint arthroplasty. There was a potential for biases in the study designs used for the eligible studies as they employed observational and interpretive designs. The findings cannot be generalised to other complications after joint arthroplasty as all of the studies focused on surgical site infection.

## Conclusions

In summary, our review shows that prosthetic joint infection can have a devastating impact on patients and that patients need more physical, psychological and social support. There is a lack of evidence on support strategies for patients undergoing treatment for PJI and other adverse events. The limited evidence available suggests that there is a worrying lack of service provision for these patients. This review highlights the need for the design, implementation, and evaluation of integrated care pathways to support patients undergoing treatment for prosthetic joint infection and other adverse outcomes following hip or knee replacement. Hip and knee arthroplasty are common interventions for the treatment of painful joint conditions and the increasing number of operations being performed means the number of prosthetic joint infections and other complications will remain significant. The development of care pathways that address the psychological, social, emotional, and economic needs of patients experiencing complications after hip and knee arthroplasty should be a research priority. Work is currently underway within our research group to develop and implement a care pathway to address the unmet needs of this patient group [31].

## Supporting Information

S1 Appendix PRISMA checklist. (DOCX)

S2 Appendix Literature search strategy. (DOCX)

S3 Appendix Reference list of excluded studies. (DOCX)
