# Supplementary material for: Health Care Needs and Support for Patients Undergoing Treatment for Prosthetic Joint Infection following Hip or Knee Arthroplasty: A Systematic Review
Source: PLoS One. 2017 Jan 3;12(1):e0169068. doi: 10.1371/journal.pone.0169068 (PMC5207523; doi:10.1371/journal.pone.0169068)
Supplement: S2 Appendix — (DOCX) [file pone.0169068.s002.docx]

**S2 Appendix.** Literature search strategy

Relevant studies, published from 1980 to February 15, 2016 (date last searched), were identified through electronic searches not limited to the English language using MEDLINE, EMBASE, Web of Science, PsycINFO, Cinahl, Social Science Citation Index, and Cochrane databases. Electronic searches were supplemented by scanning reference lists of articles identified for all relevant studies (including review articles), by hand searching of relevant journals and by correspondence with study investigators.

1 Prosthesis-Related Infections/ or periprosthetic joint infection.mp. or exp Surgical Wound Infection/ (39023)

2 exp Arthritis, Infectious/ or exp Bacterial Infections/ or prosthetic joint infection.mp. (788415)

3 joint infection.mp. or exp Osteomyelitis/ (22002)

4 exp Postoperative Complications/ or deep infection.mp. (447483)

5 wound infection.mp. or exp Wound Infection/ (47910)

6 surgical infection.mp. (760)

7 sepsis.mp. or exp Sepsis/ (143386)

8 surgical site infection.mp. (3505)

9 dislocation.mp. or exp Dislocations/ (55445)

10 fracture.mp. (156080)

11 pseudo-tumour.mp. (92)

12 exp Prosthesis Failure/ or aseptic loosening.mp. (24899)

13 septic arthritis.mp. or exp Arthritis, Infectious/ (14469)

14 adverse events.mp. (85522)

15 bone infection.mp. or exp Fractures, Open/ (5514)

16 arthroscopy.mp. or exp Arthroscopy/ (21713)

17 arthroplasty.mp. or exp Arthroplasty, Replacement/ or exp Arthroplasty, Replacement, Hip/ or exp Arthroplasty/ or exp Arthroplasty, Replacement, Knee/ (61142)

18 Hip Prosthesis/ or Joint Prosthesis/ or joint replacement.mp. (32054)

19 total joint replacement.mp. (1358)

20 hip replacement.mp. (9020)

21 total hip replacement.mp. or exp Arthroplasty, Replacement, Hip/ (22654)

22 excision arthroplasty.mp. (152)

23 debridement.mp. or exp Debridement/ (24852)

24 exp Social Support/ or exp Needs Assessment/ or support needs.mp. (78621)

25 support intervention.mp. (604)

26 support pathway.mp. (16)

27 treatment pathway.mp. (250)

28 exp Critical Pathways/ or care pathway.mp. or exp Rehabilitation Centers/ (18807)

29 service provision.mp. (4666)

30 supportive care.mp. (10415)

31 usual care.mp. (9384)

32 rehabilitation.mp. or exp Rehabilitation/ (253652)

33 exp Counseling/ or counselling.mp. (51463)

34 psychological support.mp. (2421)

35 exp Self-Help Groups/ or peer support.mp. (11088)

36 exp Education/ or education.mp. or exp "Early Intervention (Education)"/ (806653)

37 exp Physical Therapy Modalities/ or physical therapy.mp. (127781)

38 exp Exercise Therapy/ or physiotherapy.mp. (47003)

39 occupational health.mp. or exp Occupational Health/ (45469)

40 financial support.mp. or exp Financial Support/ (41181)

41 financial advice.mp. (26)

42 palliative care.mp. or exp Palliative Care/ (49718)

43 1 or 2 or 3 or 4 or 5 or 6 or 7 or 8 or 9 or 10 or 11 or 12 or 13 or 14 or 15 (1579644)

44 16 or 17 or 18 or 19 or 20 or 21 or 22 or 23 (119316)

45 24 or 25 or 26 or 27 or 28 or 29 or 30 or 31 or 32 or 33 or 34 or 35 or 36 or 37 or 38 or 39 or 40 or 41 or 42 (1320142)

46 43 and 44 and 45 (4188)

47 limit 46 to (humans and yr="1980 -Current") (3797)

Each part was specifically translated for searching the other databases (EMBASE, Web of Science, PsycINFO, Cinahl, Social Science Citation Index, and Cochrane databases)
