# Supplementary material for: Health Care Needs and Support for Patients Undergoing Treatment for Prosthetic Joint Infection following Hip or Knee Arthroplasty: A Systematic Review
Source: PLoS One. 2017 Jan 3;12(1):e0169068. doi: 10.1371/journal.pone.0169068 (PMC5207523; doi:10.1371/journal.pone.0169068)
Supplement: S3 Appendix — (DOCX) [file pone.0169068.s003.docx]

**S3 Appendix.** Reference list of excluded studies

1. Borgquist L, Nilsson LT, Lindelow G, Wiklund I, Thorngren KG. Perceived health in hip-fracture patients: a prospective follow-up of 100 patients. *Age Ageing.* 1992;21(2):109-116.

2. Biffl WL, Smith WR, Moore EE, et al. Evolution of a multidisciplinary clinical pathway for the management of unstable patients with pelvic fractures. *Ann Surg.* 2001;233(6):843-850.

3. Roberts HC, Pickering RM, Onslow E, et al. The effectiveness of implementing a care pathway for femoral neck fracture in older people: a prospective controlled before and after study. *Age Ageing.* 2004;33(2):178-184.

4. Cook JR, Warren M, Ganley KJ, Prefontaine P, Wylie JW. A comprehensive joint replacement program for total knee arthroplasty: a descriptive study. *BMC musculoskeletal disorders.* 2008;9:154.

5. Barbieri A, Vanhaecht K, Van Herck P, et al. Effects of clinical pathways in the joint replacement: a meta-analysis. *BMC Med.* 2009;7:32.

6. Berger RA, Kusuma SK, Sanders SA, Thill ES, Sporer SM. The feasibility and perioperative complications of outpatient knee arthroplasty. *Clin Orthop.* 2009;467(6):1443-1449.

7. Tian W, DeJong G, Munin MC, Smout R. Patterns of rehabilitation after hip arthroplasty and the association with outcomes: an episode of care view. *Am J Phys Med Rehabil.* 2010;89(11):905-918.

8. Beaupre LA, Masson EC, Luckhurst BJ, Arafah O, O'Connor GJ. A randomized pilot study of a comprehensive postoperative exercise program compared with usual care following primary total hip arthroplasty in subjects less than 65 years of age: feasibility, selection of outcome measures and timing of assessment. *BMC musculoskeletal disorders.* 2014;15:192.
